# Metformin dosage and galectin-3 levels: insights from PCOS patients preparing for IVF

**DOI:** 10.3389/fphar.2024.1505022

**Published:** 2025-01-31

**Authors:** Valentina N. Nikolić, Milan Stefanović, Dejan Mitić, Slavica Sunarić, Vladana Stojiljkovic, Hristina Trajković, Aleksandra Ignjatović, Dragana Stokanović

**Affiliations:** ^1^ Department of Pharmacology with Toxicology, University of Nis Faculty of Medicine, Niš, Serbia; ^2^ Department of Gynaecology and Obstetrics, University of Nis Faculty of Medicine, Niš, Serbia; ^3^ Gynaecology and Obstetrics Clinic, University Clinical Centre Niš, Niš, Serbia; ^4^ Department of Chemistry, University of Niš Faculty of Medicine, Niš, Serbia; ^5^ Department of Biochemistry, Medical Faculty University of Niš, University Clinical Center Niš, Niš, Serbia; ^6^ Department of Medical Statistics and Informatics, University of Nis Faculty of Medicine, Niš, Serbia

**Keywords:** polycystic ovary syndrome (PCOS), galectin-3, metformin, hyperprolactinemia, body mass index, metabolic pathways, insulin resistance

## Abstract

This study explores the impact of metformin dosage and hyperprolactinemia on galectin-3 levels in women with Polycystic Ovary Syndrome (PCOS), providing novel insights into their roles in the metabolic and hormonal management of the condition. A cohort of 53 women, diagnosed using the Rotterdam criteria and undergoing *in vitro* fertilization (IVF) preparation, was analyzed to determine how these factors influence galectin-3, a biomarker in PCOS. Using high-performance liquid chromatography to measure metformin concentrations and ELISA for galectin-3, our results revealed that both metformin dosage and hyperprolactinemia significantly statistically associated with galectin-3 levels, while body mass index (BMI) showed no significant association. These findings challenge prior assumptions and suggest that galectin-3 may be regulated via pathways independent of metformin pharmacokinetics. Notably, the correlation between galectin-3 levels and metformin concentration was either absent or weak after adjusting for the daily dose, indicating that treatment duration and dosage, rather than absolute drug levels, may more critically influence galectin-3. This study offers deeper insights into the role of personalized metformin dosing in managing PCOS, enhancing the understanding of metabolic and hormonal regulation in this condition, and laying the groundwork for future targeted therapies.

## 1 Introduction

Polycystic Ovary Syndrome (PCOS) affects up to 10% of women of reproductive age and is characterized by hyperandrogenism and chronic anovulation (Escobar-Morreale, 2018; Rotterdam ESHRE/ASRM-Sponsored PCOS consensus workshop group, 2004). This condition is intricately linked with significant metabolic disturbances, including insulin resistance and dyslipidemia, which elevate the risk of developing type 2 diabetes and cardiovascular diseases. These metabolic alterations, exacerbated by chronic low-grade inflammation, make insulin resistance particularly central to the pathology of PCOS, guiding therapeutic interventions such as metformin (Rudnicka et al., 2021). While recent studies have illuminated the role of metformin in enhancing endothelial function in women with PCOS (Agarwal et al., 2010), the broader impacts of this medication on additional biomarkers remain less understood. This gap in knowledge suggests the need for further investigation into how treatments like metformin affect various aspects of PCOS beyond the commonly studied parameters. Within this context, galectin-3 emerges as a biomarker of interest, offering insights into the complex interactions between insulin resistance, chronic inflammation, and PCOS (Kruszewska et al., 2022).

Galectin-3 plays a critical role in modulating trophoblast functions such as invasion and vascular formation in normal pregnancies, which are essential for successful implantation and placental development (Chen M. et al., 2022). Research indicates that galectin-3 levels increase as a normal pregnancy progresses, suggesting its protective role in maintaining gestation (Chen M. et al., 2022; Freitag et al., 2020). Conversely, in pathological conditions like gestational diabetes mellitus, altered galectin-3 levels are observed, correlating with poor pregnancy outcomes, thus marking its significance as a potential biomarker and therapeutic target (Talmor-Barkan et al., 2020; Zhang et al., 2021). Additionally, the modulation of galectin-3 is also critical in the context of PCOS, particularly for patients undergoing *in vitro* fertilization, where its regulatory roles can impact both the implantation success and the overall health of the pregnancy (Chen M. et al., 2022; Freitag et al., 2020).

Recent studies suggest that hyperprolactinemia associated with PCOS may arise from various independent etiologies rather than a direct pathophysiological link (Delcour et al., 2019). While prolactin-secreting pituitary adenomas, a common manifestation among PCOS patients, pose concerns, their presence is not universal (Delcour et al., 2019). Nonetheless, galectin-3 has been identified as a key marker in assessing the aggressiveness of these adenomas, shedding light on their potential implications within the context of PCOS (Dai et al., 2014). This connection not only underscores the significance of galectin-3 in understanding the metabolic aspects of PCOS but also highlights its potential role in managing reproductive health challenges linked to pituitary abnormalities.

Recent studies have begun to explore the interaction of metformin with galectin-3 levels. However, significant gaps remain in our understanding, particularly concerning reproductive treatments like IVF in women with PCOS. This study aims to address these gaps by investigating how both metformin dosage and hyperprolactinemia influence galectin-3 levels, potentially offering new insights into personalized treatment protocols. To ensure the relevance and applicability of our findings, our study was conducted on a real-life cohort of women preparing for *in vitro* fertilization (IVF), with minimal exclusion criteria. This approach captures a wide range of clinical scenarios, reflecting the complexities encountered in everyday management of PCOS. By doing so, we aimed to provide insights that are directly translatable to routine clinical practice.

## 2 Materials and methods

### 2.1 Design of the study and data collection

#### 2.1.1 Objective and design

Our study aimed to identify factors influencing galectin-3 levels in patients with Polycystic Ovary Syndrome (PCOS). We designed a comprehensive protocol to collect clinical, demographic, and lifestyle data, ensuring a robust analysis of potential influences on galectin-3 levels.

#### 2.1.2 Participant selection

We enrolled 53 women aged 23 to 43, diagnosed with PCOS based on the Rotterdam criteria. All participants were preparing for *in vitro* fertilization and had been undergoing metformin therapy for at least 1 month. The short protocol using recombinant gonadotropins, combined with GnRH antagonists, is a commonly recommended method for ovarian stimulation in women with PCOS undergoing IVF (Kotlyar and Seifer, 2023), as it minimizes the risk of ovarian hyperstimulation syndrome (OHSS) while offering flexibility and efficiency in ovarian response management. Data were collected in standard clinical settings as part of routine IVF preparation, without additional interventions that could skew the results. By including patients with hyperprolactinemia, we aimed to reflect the true diversity and complexity of the PCOS population undergoing IVF treatments. This inclusion helps in understanding the broader implications of metabolic and hormonal interactions in PCOS, enhancing the relevance and applicability of our findings across a wider spectrum of the PCOS community.

#### 2.1.3 Data collection

Clinical and demographic data were systematically extracted from medical records. Lifestyle habits were assessed through structured interviews with each participant, providing insights into non-clinical factors that might influence galectin-3 levels.

#### 2.1.4 Sample collection and analysis

Blood samples were collected from each participant using two separate procedures. The first blood sample was collected for routine laboratory analyses, while the second sample was specifically designated for measuring metformin concentrations and galectin-3 levels. For the latter, blood was collected in tubes containing EDTA to prevent coagulation and ensure plasma preservation. Plasma was then separated from the blood cells through centrifugation and stored at the recommended temperature until analysis. Samples were drawn just before the next scheduled dose of metformin, after participants had been on therapy for at least 1 month, to ensure that steady-state concentrations were reached and to allow for a comprehensive evaluation of the impact of metformin therapy on galectin-3 levels. This approach was crucial for accurately measuring serum galectin-3 levels and understanding the interaction between metformin and galectin-3 in the context of PCOS.

### 2.2 Measurement of plasma Galectin‐3 levels

Plasma levels of galectin-3 were quantified using an ELISA (Enzyme-Linked Immunosorbent Assay) kit provided by Elabscience Corp. (Model E-EL-H1470). This method is widely used for its high specificity and sensitivity in detecting proteins. The detection range of the galectin-3 assay was 0.16–10 ng/mL, with a sensitivity of 0.1 ng/mL, suitable for the expected concentration range in clinical samples. All procedures were performed strictly following the manufacturer’s guidelines to ensure consistency and reliability of the results. This kit was selected based on its proven accuracy and compatibility with our plasma samples, which is crucial for the validity of our findings.

### 2.3 Determination of metformin concentration

Chromatographic analysis was performed using Agilent Technologies 1,200 Series HPLC system with a DAD detector. The HPLC column was a Zorbax NH2 column (4.6 × 150 mm, 5.0 μm) and data were managed by the Agilent ChemStation program. Metformin hydrochloride certified reference material was purchased from Sigma-Aldrich (St. Louis, MO, United States). LC-MS grade acetonitrile and methanol were obtained from Honeywell Riedel-de Haen (Germany). A slightly modified method established by Mary Rebecca et al. was used for metformin analysis in plasma samples (Rebecca et al., 2019). The mobile phase was 100% acetonitrile and the flow rate was 0.725 mL/min. The column temperature was maintained at 30°C and the UV detector was set at 232 nm. The partial validation of analytical method was conducted according to ICH guidelines. The linearity was found to be over the metformin concentration range of 0.5-5.0 μg/mL with the linear regression coefficient of R2 = 0.988 for a 95% confidence level. The experimentally estimated values of limit of detection and limit of quantification were 0.15 μg/mL and 0.40 μg/mL, respectively. The within-run accuracy of the method was found to be 87%, while within-run precision expressed as the relative standard deviation was 9.0%.

The plasma samples were prepared by mixing with acetonitrile in the volume ratio 1:1 (v/v). After centrifugation and filtration through a 0.45 μm syringe filter, 20 µL of the sample was injected into the column. The metformin concentrations were calculated by the calibration curve method by using the external standard method (International Council for Harmonisation of Technical Requirements for Pharmaceuticals for Human Use, 2022). To account for differences in metformin dosing among participants, the dose-corrected metformin plasma concentration was calculated by dividing the measured plasma concentration by the corresponding daily dose (mg). This adjustment allows for the standardization of metformin levels, enabling a more accurate exploration of its pharmacodynamic effects, such as its impact on galectin-3 concentrations.

### 2.4 Ethical statement

The study protocol was reviewed and approved by the Institutional Review Boards of the University Clinical Center Nis (approval number 38212). The study adhered to the guidelines outlined in the Helsinki Declaration, emphasizing ethical principles for medical research involving human subjects. Each subject enrolled in the study received detailed information about the study protocol upon which written informed consent was obtained.

### 2.5 Statistical analysis

Data is shown as mean, standard deviation, and, for categorical variables, number and percentage. To compare data across groups, the Student’s t-test or Mann-Whitney *U*-test was used, depending on data distribution. Correlation between continuous variables was assessed according to Pearson’s correlation coefficient. Additionally, multivariate regression analysis assessed factors affecting galectin-3 and metformin concentrations. Analysis was conducted using SPSS v27.0, with statistical significance established at 0.05.

## 3 Results

### 3.1 Descriptive statistics and correlation analysis

**FIGURE 1:**
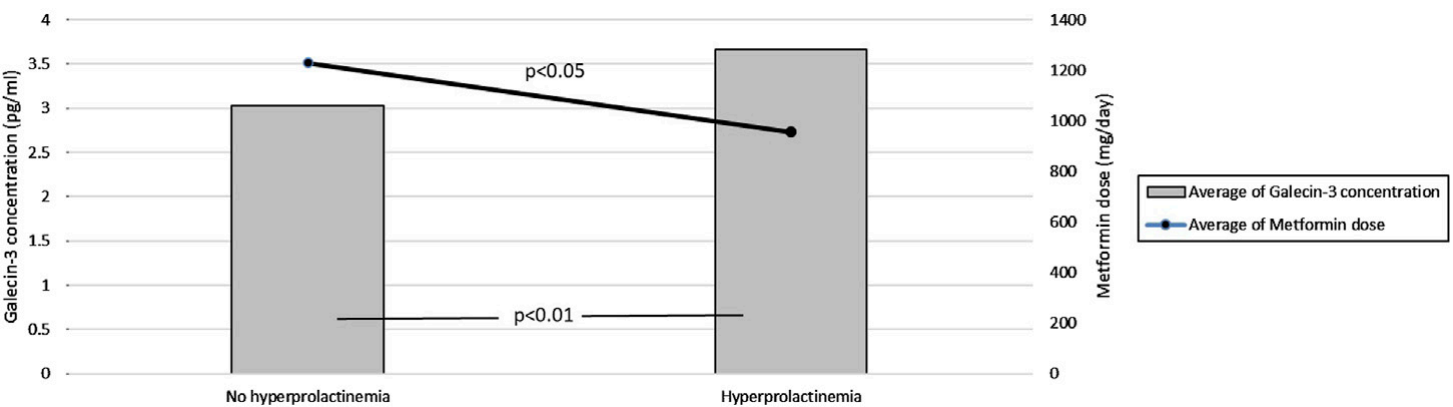
Effect of Metformin Dosage on Galectin-3 Levels in PCOS Patients with and without Hyperprolactinemia. This figure compares average galectin-3 concentrations and metformin dosages in PCOS patients with and without hyperprolactinemia. The bar heights represent mean galectin-3 levels, while the overlaid lines indicate mean metformin dosages.

Our study examined various parameters in women diagnosed with PCOS, summarized in Table 1. We noted several key relationships:

• Metformin Dosage and Galectin-3 Levels: There was a weak positive correlation between daily metformin dose and galectin-3 concentrations (r = 0.366, p < 0.05), suggesting that higher doses of metformin may modulate galectin-3 levels. Additionally, a weak negative correlation between galectin-3 concentrations and metformin concentration adjusted by the daily dose (p < 0.05) indicates complex pharmacokinetic interactions.
• Hyperprolactinemia Effects: Multivariate regression analysis, as summarized in Table 2, showed that hyperprolactinemia (Beta = 0.484, p = 0.036) and metformin dose (Beta = 0.461, p = 0.050) were significantly associated with galectin-3 levels, highlighting the influence of hormonal environment and treatment regimen. Figure 1 illustrates these findings, showing the average galectin-3 concentration and metformin dosage in PCOS patients, categorized by the presence of hyperprolactinemia. The bar heights represent the average galectin-3 levels, while the overlaid lines indicate the average metformin dosages.
• BMI Independence: Despite known influences of BMI on pharmacokinetics, BMI did not significantly affect galectin-3 levels (p = 0.991), suggesting an independent regulatory mechanism separate from typical metformin pharmacokinetics.

**TABLE 1:** Descriptive statistics of the examined parameters and laboratory findings.

Parameter	Mean ± SD (Min-Max)	Parameter	Mean ± SD (Min-Mx)
Age	32.08 ± 4.41 (23–43)	HbA1c	31.41 ± 2.82 (26.8–38.0)
Body Mass Index	23.03 ± 2.96 (19.1–30.8)	Leukocytes (109/L)	7.54 ± 2.12 (4.5–12.7)
Hyperprolactinemia (%)	11 (20.8%)	Lymphocytes (%)	31.82 ± 6.97 (16.8–47.2)
Number of patients with hypothyroidism	12 (22.6%)	Monocytes (%)	5.37 ± 1.87 (1.9–10.6)
Number of patients with hyperthyroidism	3 (5.7%)	Granulocytes (%)	65.53 ± 8.15 (53.5–80.4)
Duration of therapy (months)	12.19 ± 12.59 (1–72)	Neutrophils (109/L)	4.24 ± 1.57 (2.27–7.99)
Metformin dosage (mg)	1,166.67 ± 577.35 (500–2,500)	Erythrocytes (1012/L)	4.55 ± 0.9 (3.91–6.08)
Glucose (mmol/L)	4.93 ± 0.69 (3.9–7.3)	Hematocrit	0.40 ± 0.03 (0.33–0.47)
Urea (mmol/L)	3.80 ± 1.13 (1.5–6.4)	MCV (fL)	88.63 ± 5.16 (70.5–96.5)
Creatinine (mmol/L)	68.38 ± 10.10 (44.8–88.2)	MCH (pg)	29.03 ± 2.52 (20.1–32.8)
Albumin (g/dL)	43.50 ± 0.71 (43.0–44.0)	MCHC (g/L)	328.92 ± 12.55 (298–351)
Cholesterol (total) (mmol/L)	5.25 ± 0.95 (3.49–7.97)	RDW (%)	12.13 ± 0.94 (10.4–15.4)
HDL-C (mmol/L)	1.47 ± 0.36 (0.63–2.52)	Trombocytes (109/L)	261.06 ± 1.52 (162–485)
LDL-C (mmol/L)	3.18 ± 0.90 (1.31–5.50)	MPV (fL)	8.34 ± 1.14 (6.4–12.3)
Triglycerides (mmol/L)	1.34 ± 0.74 (0.52–4.16)	PDW (%)	18.9 ± 2.12 (15.4–21.8)
AST (U/L)	17.15 ± 4.37 (11–28)	PCT (ng/mL)	0.02 ± 0.005 (0.01–0.04)
ALT (U/L)	18.72 ± 13.08 (7–81)	Metformin (mg/L)	1.88 ± 0.28 (1.24–2.38)
CRP (mg/L)	4.20 ± 3.88 (0.3–20.5)	Galectin-3 (pg/mL)	3.16 ± 1.17 (1.55–6.12)

Abbreviations: HDL-C, high-density lipoprotein cholesterol; LDL-C, low-density lipoprotein cholesterol; AST, aspartate aminotransferase; ALT, alanine amiotransferase; CRP, C-reactive protein; MCV, mean corpuscular volume; MCH, mean corpuscular hemoglobin; MCHC, mean corpuscular hemoglobin concentration; RDW, red cell distribution width; MPV, mean platelet volume; PDW, platelet distribution width; PCT, procalcitonin.

**TABLE 2:** Multivariate regression analysis outcomes of galectin-3 levels in PCOS patients.

	Regression analysis			
	B	Se	Beta	*p*-value
Constant	4.197	2.845		0.156
Age	−0.080	0.074	−0.230	0.290
Smoking	0.185	0.603	0.077	0.763
BMI	0.002	0.138	0.004	0.991
Creatinine clearance	−0.001	0.019	−0.021	0.960
Hyperprolactinemia	1.306	0.580	0.484	0.036
Dose of metmorfin	0.001	0.001	0.461	0.050

### 3.2 Implications of findings

The complexity of galectin-3 regulation in PCOS emphasizes the need for further investigation into the metabolic and hormonal pathways influenced by these interactions. The lack of a significant direct correlation between absolute metformin concentrations and galectin-3 levels underscores the importance of metformin dosage and administration timing, suggesting the potential for personalized treatment strategies based on individual metabolic responses. Our findings are directly applicable to clinical practice as they are based on real-world data collected from patients during routine IVF preparation. The real-life approach of our study enhances the relevance of the results and provides a better understanding of how different metformin dosages and the presence of hyperprolactinemia influence galectin-3 levels, potentially leading to more personalized therapeutic strategies.

## 4 Discussion

We found a significant correlation between metformin dosage and galectin-3 levels, but no association with BMI or dose-corrected metformin plasma concentration. This suggests that the total metformin dosage plays a more critical role in modulating galectin-3 levels, highlighting its pharmacodynamic effects. Our previous study Nikolić et al. (2024) demonstrated that BMI significantly influences dose-corrected plasma concentration, underscoring the pharmacokinetic impact of BMI. However, in the current study, pharmacokinetic factors such as BMI and plasma concentration were not linked to galectin-3 modulation, suggesting that the therapeutic impact of metformin may operate through pharmacodynamic mechanisms, particularly in modulating inflammatory and metabolic pathways.

In the context of emerging research on PCOS and galectin-3 levels, our study presents a unique insight into the influence of metformin dosage and hyperprolactinemia. While previous studies indicated no significant difference in galectin-3 levels between PCOS patients and healthy controls, they did find associations with metabolic parameters like BMI, insulin levels, and HOMA-IR (Alves et al., 2020). In contrast Yilmaz et al. (2022) reported significantly higher galectin-3 levels in PCOS patients compared to controls, correlating with insulin resistance markers and hormone levels. Our findings expand on this by demonstrating a direct correlation between metformin dosage and galectin-3 levels, suggesting a dose-dependent modulation of galectin-3 in PCOS management, which has not been explicitly documented before. Moreover, we report that hyperprolactinemia significantly elevates galectin-3 levels, adding another layer to the multifaceted relationship between metabolic, hormonal, and therapeutic factors in PCOS.

Our study found that hyperprolactinemia, as indicated by bromocriptine use, was significantly associated with galectin-3 levels. However, since prolactin levels were not measured at the time of blood sampling for galectin-3 and metformin, the precise role of prolactin in modulating galectin-3 levels remains unclear. Future studies should include prolactin measurements and explore the relationship between different prolactin levels (mild vs. severe) and galectin-3 regulation to better understand how prolactin influences galectin-3 in the context of PCOS.

The potential impact of gonadotropins, particularly human chorionic gonadotropin (hCG), on galectin-3 levels during ovarian stimulation protocols is an additional factor to consider in understanding our findings. Previous studies have indicated that hCG, along with hormones such as estrogen and progesterone, can regulate galectin-3 expression, particularly in endometrial cells and trophoblastic cells, which play a role in embryo-maternal interactions (Yang et al., 2013). However, since baseline galectin-3 measurements were not taken before gonadotropin therapy in our study, we cannot confirm or exclude their contribution to the observed galectin-3 levels. This limitation emphasizes the need for further research to explore the direct effects of gonadotropin therapy on galectin-3 and its role in the metabolic and hormonal imbalances associated with PCOS.

Recent studies have broadened our understanding of the pleiotropic effects of metformin beyond its glucose-lowering properties. Metformin may modulate galectin-3 levels through the mTOR signaling pathway, a critical regulator of both metabolic processes and inflammatory responses (Chen X. et al., 2022). Galectin-3 has been implicated in mTORC1 signaling, particularly in cellular metabolism and its compartmentalization in lysosomes, suggesting that the suppression of this pathway by metformin could reduce galectin-3 expression, thereby alleviating insulin resistance and chronic inflammation in PCOS (Rudnicka et al., 2021; Chen X. et al., 2022). This hypothesis provides a molecular framework for how metformin exerts its multifaceted therapeutic effects. Further research is warranted to explore this intricate molecular interaction, particularly in the context of the role of galectin-3 in the pathophysiology of PCOS and its potential as a therapeutic target.

Additionally, the effect of metformin on mitochondrial function, particularly its interaction with mitochondrial complex I, may represent another mechanism through which it alleviates metabolic disturbances in PCOS (Foretz et al., 2023). Mitochondrial dysfunction is a hallmark of PCOS, contributing to insulin resistance and inflammation, both of which are central to the condition’s pathophysiology (Finsterer, 2023). By reducing oxidative stress and enhancing mitochondrial efficiency, metformin lowers inflammation and insulin resistance, key contributors to galectin-3 overexpression (Zhang et al., 2021; Zhu et al., 2023; Kingwell, 2016). Although the connection between mitochondrial modulation and galectin-3 appears indirect, reducing inflammation and improving insulin sensitivity may ultimately result in lower galectin-3 levels, further supporting the therapeutic effects of metformin in PCOS.

The dual action of metformin—on metabolic pathways and galectin-3 suppression—may underlie its overall therapeutic efficacy in managing PCOS. This is particularly relevant given that chronic low-grade inflammation is central to PCOS pathophysiology. By lowering galectin-3, which is associated with inflammatory signaling, metformin may improve clinical outcomes, including reproductive success (Zhu et al., 2023; Hirsch et al., 2012; Zhang et al., 2019).

Furthermore, the observation that galectin-3 levels were not significantly associated with metformin concentration in the studied population indicates that galectin-3 may be more sensitive to the dosage and administration pattern of metformin rather than its absolute concentration in the bloodstream. Additionally, our previous findings have demonstrated that BMI plays a significant role in influencing metformin plasma concentration, even when dosage is adjusted. Higher BMI was associated with lower dose-corrected metformin concentrations, potentially diminishing the drug’s efficacy in PCOS management (Alves et al., 2020). This highlights the importance of personalized dosing strategies, particularly in individuals with elevated BMI, to optimize metformin’s therapeutic effects in PCOS. This insight opens up new avenues for research into the pharmacodynamics of metformin in patients with PCOS and its interaction with galectin-3, a marker that has been increasingly recognized for its role in inflammatory processes and insulin resistance (Zhang et al., 2021; Zhu et al., 2023).

The role of hyperprolactinemia as a comorbidity in our real-life cohort offers important insights into the modulation of galectin-3 levels. Studies, such as those by Righi et al. (2010), have shown that galectin-3 is significantly upregulated in prolactin-secreting tumors, likely mediated by estrogen-related signaling pathways that influence both cellular growth and inflammation. While our cohort did not include confirmed pituitary tumors, the shared hormonal imbalances in PCOS and hyperprolactinemia suggest that similar mechanisms may contribute to elevated galectin-3 levels in these patients. The inclusion of hyperprolactinemic patients in our study strengthens the real-world applicability of our findings, but it also introduces complexities. Since galectin-3 levels were not directly correlated with metformin concentration, this may indicate a nuanced interaction between dosage, administration patterns, and the broader hormonal environment in hyperprolactinemia. Further research is warranted to disentangle these variables and better understand how hyperprolactinemia influences galectin-3 modulation in PCOS.

These findings emphasize the complexity of metabolic and hormonal regulation in PCOS and point to the importance of personalized treatment strategies. This real-life approach allowed for the inclusion of a broad spectrum of clinical scenarios, providing a realistic assessment of the impact of metformin and hyperprolactinemia on galectin-3 levels in women with PCOS. The inclusion of 11 participants receiving bromocriptine, while not confirming specific adenomas, contributes to understanding the association between hyperprolactinemia and galectin-3 levels in the context of IVF preparation. These findings emphasize the relevance of studying heterogeneous patient populations to uncover the complex interplay of metabolic and hormonal factors influencing galectin-3.

Additionally, galectin-3 inhibitors represent a promising area of exploration for future therapeutic interventions in PCOS. Recent advances in the development of galectin-3 inhibitors for other conditions, such as idiopathic pulmonary fibrosis and cardiovascular diseases, provide a rationale for investigating their application in PCOS management (Ahmed et al., 2023). Given the role of galectin-3 in driving both insulin resistance and inflammatory processes, targeting this pathway may offer new strategies for improving both metabolic and reproductive outcomes in women with PCOS (Kingwell, 2016; Yan et al., 2020).

Furthermore, an in-depth exploration of metformin’s pharmacodynamics, particularly its interaction with galectin-3 in the presence of hyperprolactinemia, is critical. By advancing our understanding of these mechanisms, we could pave the way for more targeted, personalized treatment approaches that address the complex hormonal and metabolic dysregulation in PCOS.

### 4.1 Clinical implications

**FIGURE 2:**
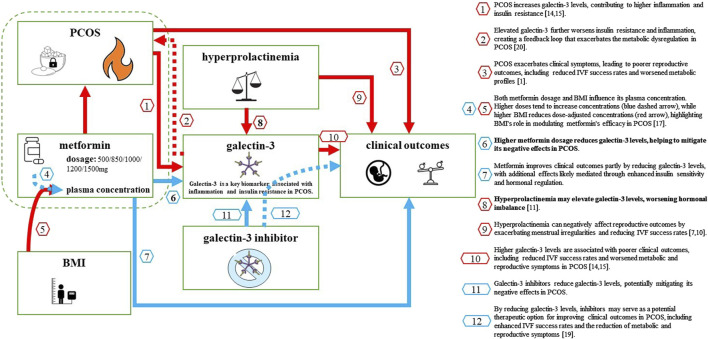
Key Factors Influencing Galectin-3 Levels in Women with PCOS and Their Impact on Clinical Outcomes. This diagram illustrates the complex relationships between factors that influence galectin-3 levels in women with PCOS and their impact on clinical outcomes. PCOS increases galectin-3 levels by elevating inflammation and insulin resistance (red arrow 1) and exacerbates clinical symptoms, leading to poorer reproductive outcomes (red arrow 2). Metformin, at higher dosages, reduces galectin-3 levels (blue arrow 3) and improves clinical outcomes both by lowering galectin-3 and through additional pathways, including enhanced insulin sensitivity (blue arrow 4). BMI also plays a significant role by reducing dose-corrected metformin plasma concentrations (red section of arrow 6), while higher metformin doses increase plasma concentrations (blue section of arrow 5), illustrating the need for personalized dosing in PCOS treatment. Hyperprolactinemia further elevates galectin-3 levels (red arrow 7) and negatively impacts reproductive outcomes (red arrow 8). Finally, potential Galectin-3 inhibitors could reduce galectin-3 levels (red arrow 10), indirectly improving clinical outcomes (dashed blue arrow 11). Fire icon: Represents inflammation, a key factor driving increased galectin-3 levels in PCOS; Sugar cube with a keyhole: Symbolizes insulin resistance, another major contributor to elevated galectin-3 levels; Unbalanced scales: Illustrates hormonal imbalance associated with hyperprolactinemia, affecting galectin-3 levels in certain patients; Crossed molecular structure icon: Represents the potential therapeutic galectin-3 inhibitor, a molecule that specifically blocks or reduces galectin-3 levels, which could help alleviate inflammation, improve insulin sensitivity, and enhance reproductive outcomes in women with PCOS. Arrow colours: Blue arrows indicate positive effects, where interventions like metformin or galectin-3 inhibitors contribute to better clinical outcomes or reduce galectin-3 levels; Red arrows indicate negative impacts, such as factors like PCOS and BMI increasing galectin-3 levels or reducing metformin efficacy.

Our findings suggest that metformin dosage, rather than plasma concentration or BMI, plays a key role in modulating galectin-3 levels in women with PCOS. This insight highlights the importance of personalized metformin dosing strategies, particularly in patients with elevated BMI, where pharmacokinetic factors may reduce the drug’s efficacy. Given the potential for galectin-3 to serve as a marker of both metabolic dysfunction and inflammation, targeting galectin-3 with inhibitors—alongside optimized metformin therapy—could provide a promising approach for improving clinical outcomes in PCOS, including metabolic and reproductive health. Large-scale clinical trials are essential to validate the use of galectin-3 inhibitors as a therapeutic strategy for PCOS (Figure 2).

### 4.2 Limitations

While our study offers valuable insights, it is limited by the relatively small sample size and cross-sectional design, which may affect the generalizability of the findings. Additionally, our reliance on real-world data introduces some variability in patient characteristics, particularly in terms of metformin adherence and BMI distribution. Importantly, androgen levels were not assessed in this study, precluding us from investigating potential correlations with galectin-3 levels. Despite these limitations, our findings provide a foundation for future research to explore the pharmacodynamics of metformin and galectin-3 interactions in a larger, more controlled cohort. Further studies should also assess the long-term effects of metformin dosage on galectin-3 modulation and its clinical outcomes in PCOS.

## 5 Conclusion

Our study provides important insights into the modulation of galectin-3 levels by metformin dosage and hyperprolactinemia in women with PCOS, highlighting the relevance of patient-specific treatment strategies for addressing the complex metabolic and hormonal challenges of this condition. By demonstrating the critical role of metformin dosage and hormonal factors, particularly hyperprolactinemia, in modulating galectin-3 levels, these findings challenge traditional paradigms focused solely on pharmacokinetics and pave the way for exploring galectin-3 as a novel therapeutic target in PCOS.

The potential clinical application of galectin-3 inhibitors, in conjunction with personalized metformin dosing, represents a promising therapeutic avenue. Future research should focus on large-scale clinical trials to validate the utility of galectin-3 as both a biomarker and a therapeutic target. Additionally, exploring the pharmacodynamics of metformin in relation to galectin-3 will be crucial for advancing PCOS treatment protocols and optimizing patient outcomes.

While our study provides valuable insights, further research with larger cohorts is needed to confirm these findings and explore the broader implications of galectin-3 modulation in PCOS.

## Data Availability

The raw data supporting the conclusions of this article will be made available by the authors, without undue reservation.
